# Esketamine versus fentanyl as adjuncts to hepatic hilar nerve block for ambulatory percutaneous liver tumor ablation focusing on respiratory safety: protocol for a randomized controlled trial

**DOI:** 10.3389/fphar.2025.1644029

**Published:** 2025-09-17

**Authors:** Xiao Wang, Lijuan Yan, Jiaying Cai, Jianfei Wei, Zuobing Zhang, Bin Yang

**Affiliations:** ^1^ Department of Ultrasound, The First Affiliated Hospital of Xiamen University, School of Medicine, Xiamen University, Xiamen, China; ^2^ Department of Anesthesiology, The First Affiliated Hospital of Xiamen University, School of Medicine, Xiamen University, Xiamen, China; ^3^ Department of Scientific Research, The First Affiliated Hospital of Xiamen University, Xiamen, China; ^4^ Department of Anesthesiology, The First Hospital of Zhangzhou China Merchants Economic and Technological Development Zone, Zhangzhou, China

**Keywords:** esketamine, thermal ablation, hepatic hilar nerve block, ambulatory anesthesia, opioid-sparing analgesia

## Abstract

**Background:**

Opioid-induced respiratory depression (OIRD) is a critical safety concern during ambulatory percutaneous liver tumor thermal ablation. Esketamine has been shown to offer a promising opioid-sparing alternative with the potential to provide respiratory stability benefits. We hypothesize that hepatic hilar nerve block (HHNB) combined with esketamine will reduce the incidence of respiratory depression when compared to HHNB in conjunction with fentanyl in this particular context.

**Methods:**

This single-center, prospective, double-blind, randomized controlled trial (RCT) will enroll patients undergoing ambulatory ultrasound-guided percutaneous liver thermal ablation. Patients will be randomly assigned to receive either intravenous esketamine 0.37 mg kg^−1^ (Intervention group) or intravenous fentanyl 1 μg·kg^−^ (Control group). All subjects will receive a standardized premedication consisting of midazolam 0.03 mg kg^−1^ IV, followed by ultrasound-guided HHNB.

**Results and analysis:**

The primary outcome is the incidence of respiratory depression, defined as SpO_2_ <90% or EtCO_2_ >55 mmHg. Secondary outcomes include the rate of anesthesia success, postoperative pain scores, and the consumption of remedial analgesia at 2, 6, and 24 h post-surgery. Additionally, satisfaction scores from both the sonographer and the patient are considered, along with any adverse events that may occur. The statistical analysis will utilize appropriate parametric/non-parametric tests for continuous data and chi-square/Fisher’s exact tests for categorical data (significance p < 0.05), using SPSS (v20.0) and R (v4.4.3; R Foundation) within the RStudio environment (v2024.12.1 + 563).

**Conclusion and discussion:**

This trial aims to provide Level I evidence comparing the respiratory depression risk between esketamine-based and fentanyl-based analgesia during HHNB-guided liver ablation. Should esketamine prove to be demonstrably superior in terms of respiratory safety, HHNB-esketamine has the potential to be a viable treatment option.

## 1 Introduction

Percutaneous thermal ablation is a minimally invasive treatment for early-stage hepatocellular carcinoma and oligometastatic liver disease. Its application in ambulatory settings is expanding rapidly due to advantages in patient recovery and healthcare resource utilization (Shamim et al., 2017; He et al., 2021; Georgiades et al., 2025). However, providing effective and safe analgesia for these procedures remains a critical challenge in Ambulatory Surgery Centers (ASCs) or interventional centers (He et al., 2021; Georgiades et al., 2025). This is particularly true for the thermal ablation of subcapsular and exophytic liver lesions, or tumors adjacent to the parietal peritoneum (Wah et al., 2005; Lee et al., 2009). In these settings, procedural sedation and analgesia (PSA) is typically administered by the interventional team without direct anesthesiologist involvement (He et al., 2021; Georgiades et al., 2025).

The hepatic hilar nerve block (HHNB) has been introduced more recently as a complementary regional technique to enhance visceral analgesia with higher liver-specificity compared to celiac or paravertebral blocks. However, HHNB alone is often insufficient to provide complete analgesia during the procedure (He et al., 2021; Georgiades et al., 2025). Systemic opioids remain the traditional foundation for managing visceral pain, yet they carry a well-established, dose-dependent risk of opioid-induced respiratory depression (OIRD), which is mediated by µ-opioid receptors in the brainstem (Watkinson et al., 2002; Jonkman et al., 2018; Gali et al., 2019). OIRD represents a potentially life-threatening event in an ambulatory setting (Baldo and Rose, 2022), impairing both ventilation and CO_2_ responsiveness (Olsen et al., 2024). This risk is further exacerbated during liver ablation due to factors such as patient positioning, artificial ascites, and concomitant sedation administration, increasing the likelihood of hypoxemia, emergent airway interventions, and unplanned hospital admissions (Gali et al., 2019). Moreover, respiratory drive is further compromised as opioid sensitivity varies under different neurological and physiological states (Ramirez et al., 2021). Therefore, an opioid-free anesthesia (OFA) strategy (McLott and Stahel, 2022) combined with HHNB presents a highly desirable alternative for enhancing respiratory safety during ultrasound-guided percutaneous liver thermal ablation in ASCs (Liu et al., 2020).

Esketamine (S-ketamine), a potent N-methyl-d-aspartate (NMDA) receptor antagonist, not only manages treatment-resistant depression but also offers a mechanistically distinct approach to analgesia and sedation (Martinotti et al., 2022; Song et al., 2023; Di Vincenzo et al., 2024; Rosso et al., 2025). Its pivotal benefit lies in the capacity to provide analgesia and sedation while maintaining breathing and hemodynamic stability (Jonkman et al., 2018; Li et al., 2022; Miao et al., 2024). Crucially, it may stimulate breathing through NMDA receptor blockade (Jonkman et al., 2018). This distinctive pharmacological profile positions it as a highly promising opioid-sparing or opioid-free alternative, potentially mitigating the principal safety concern—OIRD in the ambulatory setting. To ensure clinical relevance and comparability, the proposed esketamine dose (0.37·mg kg^−1^ IV) in the present study protocol has been established with careful consideration. This estimate is derived from our previous Dixon sequential allocation study, conducted within the same clinical context. That study established the median effective dose (ED50) and the 95% effective dose (ED95) as 0.35 mg·kg^−1^ and 0.37·mg kg^−1^, respectively, for effective procedural analgesia (defined as no significant movement). The manuscript reporting these findings is currently under peer review.

Despite this promising profile and a defined effective dose, a critical evidence gap remains. There is a lack of high-quality randomized controlled trials (RCTs) directly comparing the incidence of respiratory depression (the most consequential safety endpoint) between an esketamine-based regimen (HHNB + esketamine 0.37 mg·kg^−1^) and the opioid-based regimen (HHNB + fentanyl 1 μg·kg^−1^) in patients undergoing ambulatory liver thermal ablation. Therefore, we designed this prospective, single-center, double-blind, randomized controlled trial (RCT) to test the hypothesis that the esketamine-based regimen is associated with a significantly lower incidence of respiratory depression.

## 2 Methods

### 2.1 Study design

**FIGURE 1:**
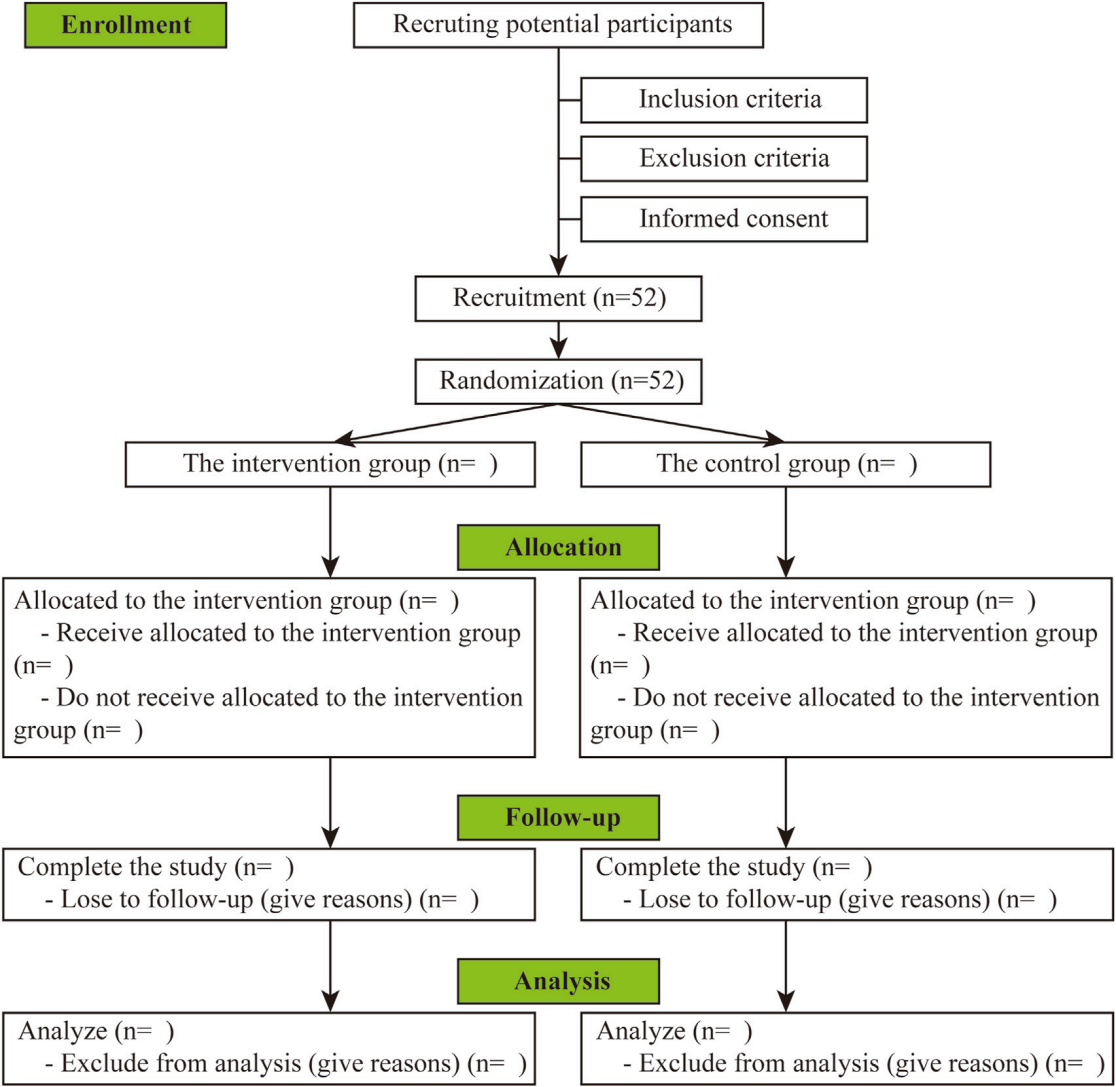
Flow chart of the study.

This RCT will be carried out in the department of Ultrasound at the First Affiliated Hospital of Xiamen University. The present protocol has been designed under the principles of the Declaration of Helsinki and is consistent with the Standard Protocol Items (Chan et al., 2013). The study flow chart is summarized in Figure 1, and a detailed schedule of enrolment, intervention, and assessments is provided in Table 1.

**TABLE 1:** The schedule of enrollment, interventions, and assessments**.** PACU: post-anesthetic care unit, NRS: Numeric Rating Scale. T1: Prior to anesthesia (10 min after positioning), T2: 60 s after esketamine injection, T3: 5 min after the hepatic hilar nerve block, T4: During thermal ablation of liver tumors, T5: In the PACU, T6: 2 h after the procedure, T7: 6 h after the procedure, T8: 24 h after the procedure.

Study period	Screening	Allocation	Intervention and procedure	PACU	Follow-up		
		T1	T2, T3, T4	T5	T6	T7	T8
Enrollment:							
Inclusion criteria	X						
Exclusion criteria	X						
Informed consent	X						
Randomization	X						
Demographic data	X						
Physical examination data	X						
Interventions:							
The intervention group			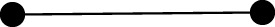				
The control group			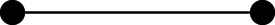				
Assessments:							
Clinical monitoring							
Vital signs		X	X	X			
NRS		X	X	X			
Outcome assessment							
The incidence of respiratory depression			X	X			
The success rate of anesthesia			X				
The consumption of remedial analgesia					X	X	X
Induction duration			X				
Awakening duration			X	X			
The detained time in hospital		X	X	X	X	X	X
Sonographer satisfaction score				X			
Patient satisfaction score							X
Adverse events			X	X	X	X	X

All participants will receive a standardized premedication consisting of midazolam at a dose of 0.03 mg·kg^-1^ and will undergo ultrasound-guided HHNB. They will be randomly assigned to either the intervention group or the control group. The intervention group will receive an intravenous infusion of esketamine at a dose of 0.37 mg·kg^−1^, while the control group will receive an intravenous infusion of fentanyl at a dose of 1 μg·kg^−1^. Subsequently, the subjects will undergo ultrasound-guided percutaneous hepatic thermal ablation under sedation. Following the procedure, patients are transferred to the PACU.

### 2.2 Timeline and assessments

Key time points for physiological data collection are as follows: T1: Prior to anesthesia (10 min after positioning); T2: 60 s after esketamine injection; T3: 5 min after HHNB; T4: During thermal ablation of liver tumors; and T5: In the PACU.

Postoperative pain assessments (for providing remedial analgesia) will be conducted at T6: 2 h, T7: 6 h, and T8: 24 h after the procedure. These time points will be chosen to capture the analgesic requirements during the early recovery phase.

### 2.3 Study sample

#### 2.3.1 Participant enrollment

The identification and enrolment of subjects suitable for ultrasound-guided percutaneous hepatic thermal ablation will take place at the outpatient clinic of the Department of Ultrasound from December 2025 to June 2028. The department is responsible for handling a yearly volume of over 100 patients who undergo ultrasound-guided percutaneous hepatic thermal ablation. Before undergoing the procedure, patients will undergo a comprehensive evaluation and will be informed of the potential benefits and common complications. Before enrolment, written informed consent will be obtained from the patient. It is imperative to note that all participants are entitled to withdraw from the study at any time. In the event of patient refusal to participate in the study, or in the event of suspension or withdrawal from the study midway, this will be completed following the alternative anesthetic plan for subsequent treatment. The quality of medical care will not be affected by any of these eventualities.

#### 2.3.2 Participants’ inclusion criteria

All subjects deemed eligible will be subjected to a screening process based on the following inclusion criteria.

• Aged 18–70 years;
• ASA physical status I or III;
• Body mass index (BMI) 18–28 kg·m^-2^;
• Scheduled for elective ultrasound-guided thermal ablation of solitary liver tumors under HHNB.

#### 2.3.3 Participants’ exclusion criteria

The following section outlines the exclusion criteria that will be applied to participants in this study.

• Known hypersensitivity to study medications (esketamine, midazolam);
• Opioid or benzodiazepine dependence;
• Using analgesics within the last 24 h preoperatively;
• Participation in other investigational drug trials within 90 days.
• Multifocal hepatic lesions requiring concurrent ablation;
• Patients after liver transplantation;
• Active upper respiratory tract infection within 14 days;
• Severe cardiopulmonary diseases (New York Heart Association [NYHA] class III-IV, FEV_1_/FVC <70%);
• Decompensated hepatic insufficiency (Child-Pugh C);
• Uncontrolled hypertension (≥180/110 mmHg), elevated intracranial/intraocular pressure, or hyperthyroidism;
• Major neuropsychiatric disorders (epilepsy, schizophrenia, major depressive disorder, and cognitive impairment).
• Anticipated difficult airway (Mallampati III-IV, thyromental distance <6 cm) or anatomical airway obstruction;
• Inadequate preoperative fasting (solid intake <8 h, clear fluids <2 h).

#### 2.3.4 Participants’ withdrawal criteria

Participants will be withdrawn from the study if any of the following conditions apply:

• Voluntary withdrawal of consent.
• Significant deviation from established anesthesia or surgical protocols.
• Concomitant surgical interventions that alter study parameters.

### 2.4 Sample size

As demonstrated in earlier research, the incidence of respiratory depression in the intervention group was found to be approximately 0.01, while in the control group, it was 0.3, with α = 0.05 and β = 0.1. The sample size was calculated to be 23 cases using R (v4.4.3; R Foundation) within the RStudio environment (v2024.12.1 + 563). In light of the observed dropout rate, estimated at approximately 10%, the sample size was determined to be 52 cases.

### 2.5 Randomization

The present study adopted a simple randomization procedure. The randomization process utilizes computer-generated random numbers, and the allocation ratio of patients is 1:1. The grouping information was sealed and stored by the research assistants, who were not involved in the study, in opaque envelopes according to the grouping numbers. Access to the randomization code is limited to specific researchers responsible for preparing the research drug and handling emergencies during the process to ensure the safety of the participants.

### 2.6 Blinding

In the design and implementation of the current study, the researchers, subjects, and followers had no knowledge of the intervention and control drugs, will be unaware of the grouping of the cases and whether the drug used is esketamine or fentanyl, and remained blinded throughout.

In this experiment, esketamine and fentanyl will be administered by a research assistant who will not be involved in the design and implementation of the study. The same syringes will be utilized for each injection, and the specified dose of the drug according to the study protocol and grouping allocation will be diluted to the same volume (20 mL) with 0.9% normal saline. The syringes will be labeled with unique identifiers and assigned to the attending anesthesiologist. The anesthesiologist will perform the anesthesia following the stipulated instructions in the envelope.

The patient will be rendered blind for the duration of the present study, as they will be in a sedated state and unaware of medical information.

The designated follow-up personnel will undertake the requisite follow-up and record data accordingly. The subsequent results should be uploaded to the data center and must not be disclosed to anyone.

### 2.7 Intervention

**FIGURE 2:**
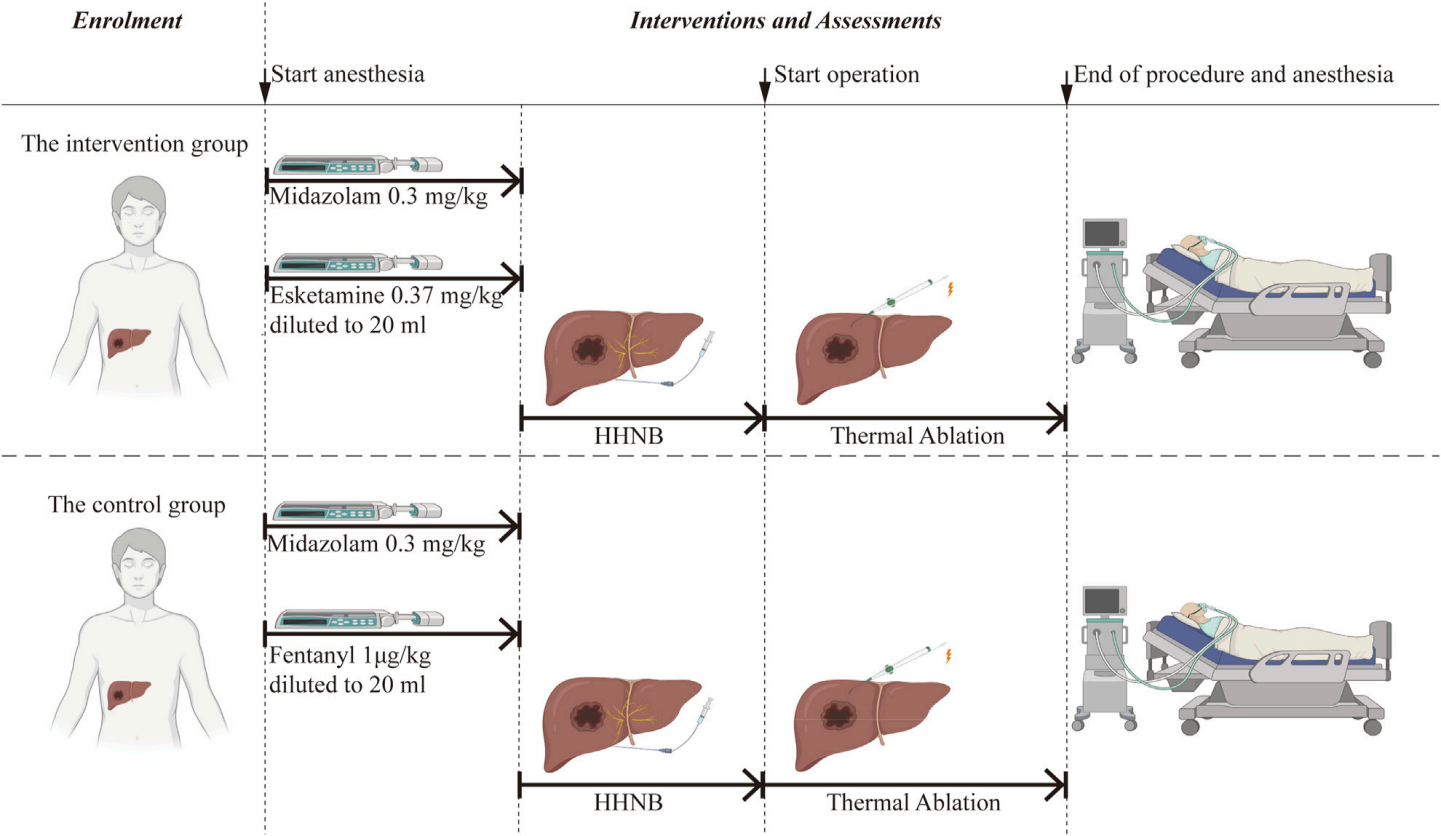
Graphic abstract. HHNB: hepatic hilar nerve block.

Once the participant has been identified, a schedule for the intervention will be established based on group allocation. The schedule of enrolment, interventions, and assessments is shown in Table 1 and Figure 2 (Graphic Abstract).

All patients will be consciously sedated using midazolam combined with esketamine/fentanyl before undergoing ultrasound-guided HHNB. The intervention group will be administered midazolam at a dose of 0.03 mg·kg^-1^ and esketamine at a dose of 0.37 mg·kg^-1^, while the control group will receive midazolam at a dose of 0.03 mg kg^-1^ and fentanyl at a dose of 1 μg·kg^-1^. Esketamine or fentanyl will be diluted to 20 mL with 0.9% normal saline and injected intravenously within 60 s. Subsequently, the ultrasound-guided percutaneous HHNB technique will be performed under the detailed description that follows.

### 2.8 Monitoring and standard practice-based anesthesia protocol

Standardized preoperative fasting protocols will be strictly enforced, requiring abstinence from solid intake for ≥8 h and clear fluids for ≥2 h before the procedure. Upon transfer to the operating room, patients will be positioned supine and instrumented with standard monitoring. Oxygen supplementation will be administered through a Venturi mask at a fixed flow rate of 5 L·min^-1^ to maintain peripheral capillary oxygen saturation (SpO_2_) ≥95% throughout the procedure. A validated anesthesia information management system will be employed for high-resolution data acquisition, capturing hemodynamic and respiratory variables at 5-s intervals. Automated artifact detection algorithms will filter spurious signals, with waveform validity confirmed by two independent anesthetists blinded to procedural timing.

**FIGURE 3:**
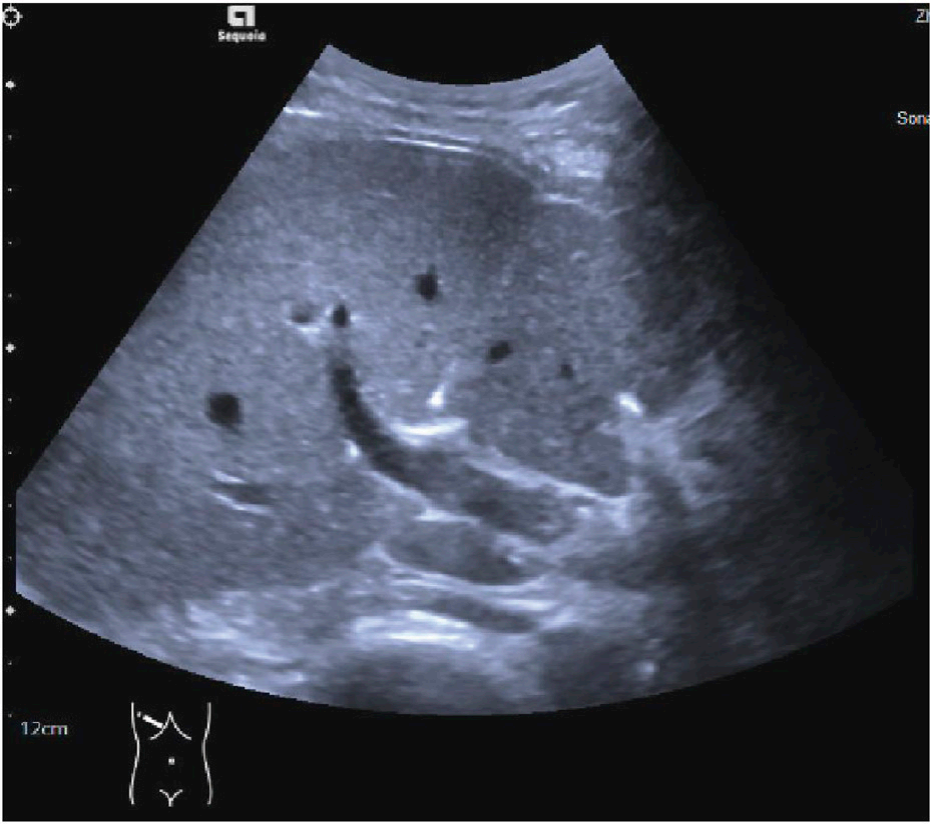
The 22G puncture needle guided to puncture the Glisson’s capsule in the grayscale mode.

**FIGURE 4:**
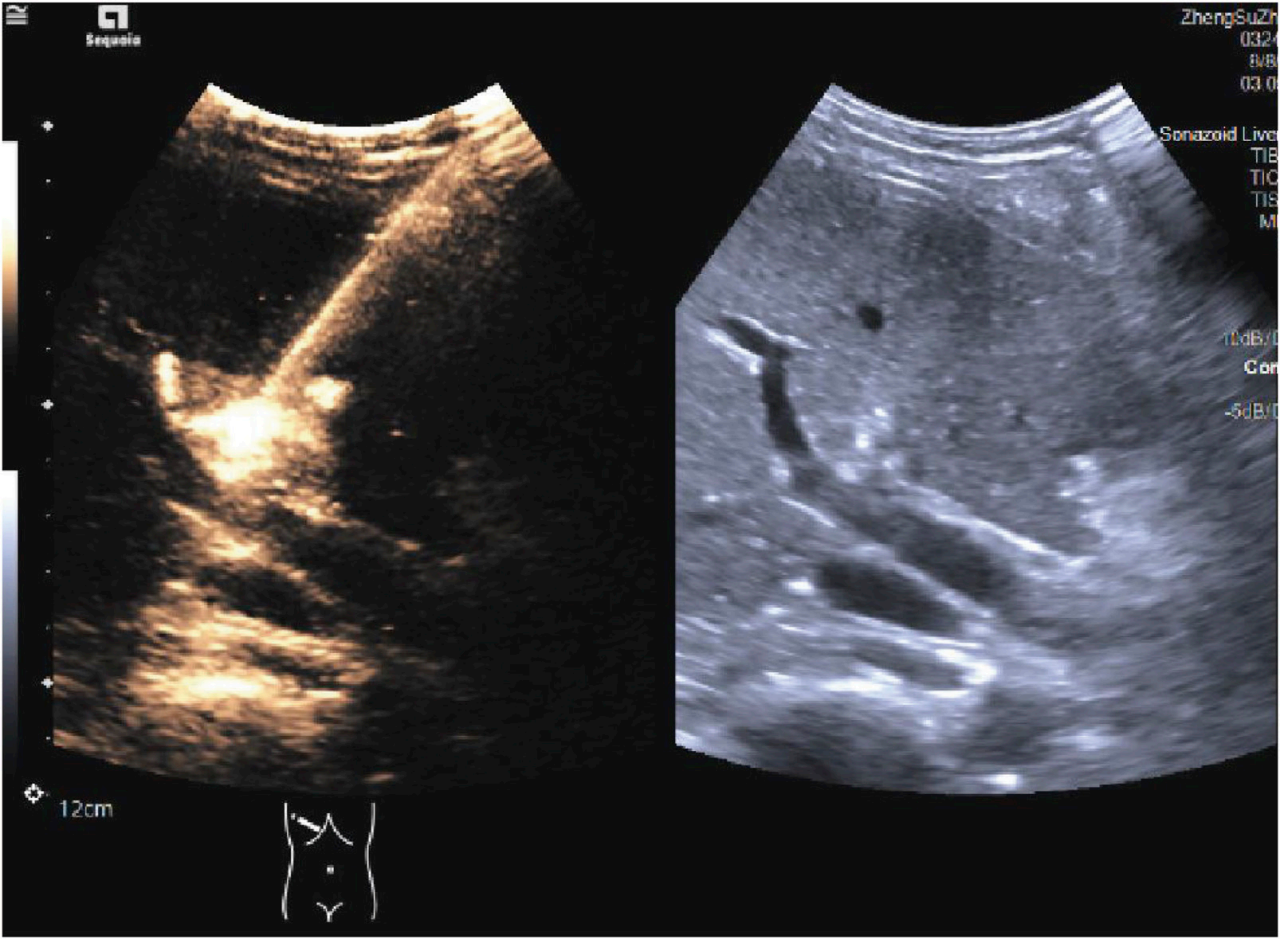
Ultrasound-guided HHNB. HHNB: hepatic hilar nerve block.

**FIGURE 5:**
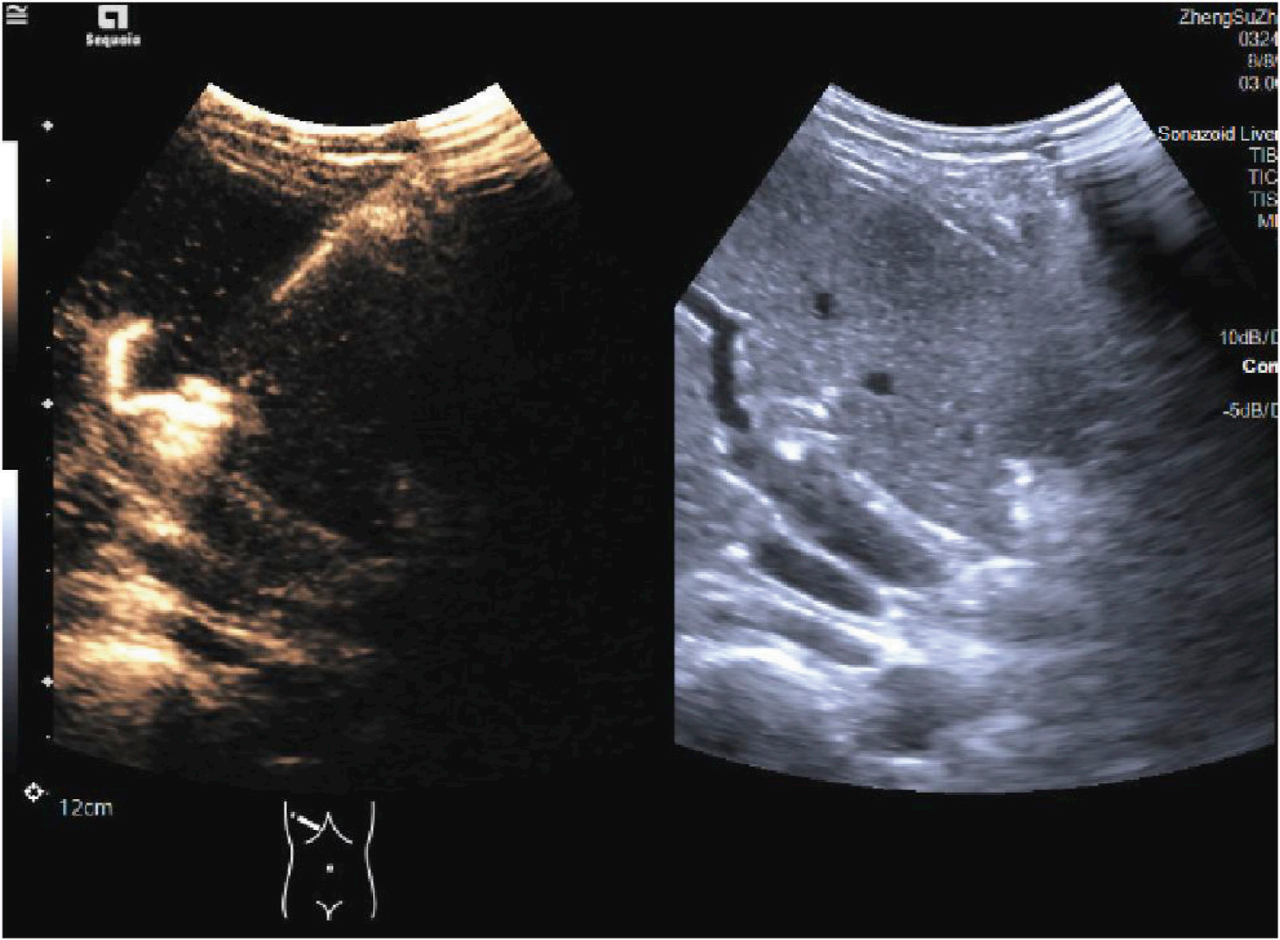
Ultrasound imaging of Glisson’s capsule after the administration of ultrasound-guided HHNB in dual-screen Doppler mode. HHNB: hepatic hilar nerve block.

*Ultrasound-guided Percutaneous HHNB Technique* Ultrasound-guided HHNB will be performed by the same board-certified ultrasound physician. Artificial ascites enhances ultrasound visualization for hepatic nerve blocks and liver tumor thermal ablation, while preventing thermal injury to adjacent vital organs (Wang and Kao, 2015; Bhagavatula et al., 2017). The induction of artificial ascites will be achieved by the injection of 1500 mL of normal saline into the abdominal cavity, via a puncture needle that will penetrate the abdominal wall. Under real-time ultrasound guidance with a 2–5 MHz curvilinear transducer, the hepatic hilum will be systematically interrogated to identify the main portal vein, hepatic artery, and bile duct using B-mode and color Doppler imaging. Following standard aseptic preparation, a skin wheal of 1% lidocaine will be administered at the premarked subcostal entry site. The trajectory of the needle will be optimized through the utilization of a trans-left hepatic approach, whereby the transducer will be angled obliquely in the subcostal plane to facilitate visualization of the portal bifurcation. A 22-gauge Chiba needle will be advanced under continuous ultrasound visualization through the left hepatic parenchyma towards the anterior aspect of the main portal vein, maintaining a safety margin of 1.5–2 cm distal to the bifurcation (He et al., 2021) (Figure 3; Supplementary Video S1). Following the aspiration test, which yielded a negative result, 0.1 mL of microbubble contrast agent (SonoVue^®^) diluted in 5 mL of saline will be administered. This will be followed by contrast harmonic imaging (CHI; MI 0.08-0.12) to verify the perivascular distribution along Glisson’s sheath (Figure 4; Supplementary Video S2). Thereafter, 10 mL of a 0.5% ropivacaine premixed with 0.1 mL of SonoVue^®^ will be delivered in 2 mL aliquots and dynamically monitored using dual-screen Doppler overlay to ensure the distribution of local anesthetic along the branch and main portal vein (Liu et al., 2020) (Figure 5; Supplementary Video S2). 15 min later, procedural commencement will be initiated.

Each rescue dose of remifentanil will be 0.5 μg·kg^-1^, based on ideal body weight, administered when inadequate analgesia and as an intravenous bolus repeated at 2-min intervals at least. Anesthesia failure will be defined as poor pain control, requiring more than three rescue doses within 10 min. In such cases, the induction of general anesthesia will be promptly facilitated by intravenous administration of propofol (100 mg), followed by the insertion of a laryngeal mask and subsequent initiation of mechanical ventilation.

Respiratory compromise management will also follow a stepwise protocol: respiratory depression (SpO_2_ <90% for >30 s or end-tidal carbon dioxide [EtCO_2_] >55 mmHg) will initiate chin-lift/jaw-thrust maneuvers progressing to positive-pressure ventilation via a facemask with 100% oxygen. Refractory hypoventilation (>60 s unresponsive to manual ventilation) will mandate supraglottic airway placement.

Bradycardia (heart rate [HR] <45 bpm) will be treated with atropine 0.5 mg intravenously. Hypotension (mean arterial pressure [MAP] <80% baseline) will trigger the administration of phenylephrine 50 μg intravenous boluses, repeated every minute until the target MAP is achieved.

All intraoperative events will be subjected to blind adjudication and timestamped by an independent research assistant. The onset duration, severity grading, and intervention frequency will be documented using case report forms.

### 2.9 Data collection, handling, and monitoring

All patient and procedure-related variables will be systematically extracted from electronic medical records using a standardized case report form (CRF). The following variables will be included: (i) Baseline characteristics of the subjects: age, sex, height, weight, ASA status; (ii) Data about the primary and secondary outcomes; (iii) Standard Monitoring: electrocardiographic (ECG), MAP, SpO_2_, respiratory rate (RR), EtCO_2_; (iv) Intraoperative events: the onset duration, severity grading, and intervention frequency.

All adverse events (AEs) will be meticulously documented in the source file and actively monitored until such events are resolved or stabilized. Serious adverse events (SAEs) and suspected and unexpected serious adverse reactions (SUSARs) must be reported within 24 h.

It is imperative to ensure that all information is accurate, complete, and submitted on time. Meanwhile, data records must be clear and comprehensible to ensure accurate interpretation and facilitate traceability.

## 3 Outcomes

The primary outcome is the incidence of respiratory depression, which is defined as SpO_2_ <90% or EtCO_2_ >55 mmHg. The second outcomes are the success rate of anesthesia, postoperative pain score, the consumption of remedial analgesia, induction duration, awakening duration, the detained time in the hospital, sonographer satisfaction score, patient satisfaction score, and adverse events.

Anesthesia success will be defined as inadequate analgesia requiring ≤3 rescue doses of remifentanil within 10 min throughout the procedure. The success rate of anesthesia will be calculated by dividing the number of successful anesthesia cases by 26.

The Numeric Rating Scale (NRS) will be utilized to assess postoperative pain, with a range of 0 representing no pain and 10 representing severe pain. This scale will be administered at 2, 6, and 24 h following the procedure.

The consumption of remedial analgesia will be the total consumption of acetaminophen documented at 2, 6, and 24 h after the procedure.

The satisfaction score of the sonographer will be collected post-operatively, using a scale ranging from 0 to 10, with 0 representing dissatisfaction and 10 representing very satisfied. The patient’s satisfaction will be assessed on the first day after the procedure (Yan et al., 2024).

Adverse events will encompass a range of systems, including the cardiovascular system (eg. high/low blood pressure and sinus tachycardia/bradycardia), as well as symptoms such as nausea, vomiting, dizziness, and mental symptoms.

## 4 Statistical analysis

All statistical analyses will be conducted using SPSS Statistics (v20.0; IBM Corp) and R (v4.4.3; R Foundation) within the RStudio environment (v2024.12.1 + 563), under the supervision of an independent biostatistician. The last observation carried forward method will be used to impute missing data, and the interim analyses will not be performed in this study. Continuous variables will undergo a process of normality assessment by the Kolmogorov-Smirnov test. In the case of normally distributed data, the following statistical procedures are to be employed: the data will be expressed as the mean ± standard deviation and analyzed with independent samples t-tests for between-group comparisons, paired t-tests for within-group assessments, and repeated-measures analysis of variance ANOVA for longitudinal measurements. In the event of non-normally distributed data, this will be reported as the median [interquartile range] using the Mann-Whitney U test for intergroup and the Wilcoxon signed-rank test for intragroup comparisons. Categorical variables will be summarized as frequencies (%), and these will be compared via chi-square tests or Fisher’s exact tests. A p-value of less than 0.05 will be considered statistically significant.

## 5 Discussion

This RCT protocol addresses a critical safety gap in ambulatory percutaneous liver tumor ablation by directly comparing the incidence of respiratory depression–the paramount anesthesia-related adverse event in ASCs–between two mechanistically distinct analgesic strategies: hepatic hilar nerve block (HHNB) augmented with either esketamine (0.37 mg·kg^-1^) or fentanyl (1 μg·kg^-1^). In contrast to opioid receptor agonism, which has been shown to decrease the hypoxic ventilatory response as well as the ventilatory response to hypercarbia (Palkovic et al., 2020), esketamine has been observed to preserve the hypoxic ventilatory response to hypoxia (Jansen et al., 2025). This mechanistic synergy is of particular relevance in the context of artificial ascites and supine positioning during ablation, where even minor respiratory compromise can escalate to hypoxemia or the need for airway intervention.

In the event of confirmation of our hypothesis, the clinical implications will extend beyond statistical significance. The mitigation of respiratory depression can predictably reduce the cascade of ambulatory care disruptions, including the administration of naloxone, emergent airway management, prolonged PACU stays, unplanned admissions, and additional medical burdens (Gali et al., 2019). Moreover, the administration of multimodal anesthesia and effective pain management has been demonstrated to reduce the utilization of opioids, decrease the incidence of postoperative respiratory depression in the PACU, and further mitigate the subsequent occurrence of serious adverse events following surgery (Laporta et al., 2021). These elements are widely regarded as the cornerstones of sustainable outpatient ablation programs.

### 5.1 Strengths of the study

The methodological strengths of the trial enhance the validity of its findings. (i) In contrast to studies utilizing arbitrary and unvalidated “low-dose” esketamine regimens, the present study employed a dose-finding trial within the same clinical context to determine the esketamine dosage for the intervention group, thereby ensuring analgesic efficacy comparable to that of the standard fentanyl protocol. (ii) The process of double-blinding serves to minimize performance and detection bias, while the utilization of an active-controlled design ensures the maintenance of clinical relevance. The utilization of continuous respiratory monitoring through the implementation of standardized capnography and oximetry techniques facilitates the objective assessment of the primary endpoint.

### 5.2 Limitations

It is imperative to acknowledge the limitations of this study. (i) The single-center design is predicated on the principle of internal validity, with the potential consequence of diminished generalizability. Nevertheless, the rigorous standardization of the HHNB technique, anesthetic protocols, surgical procedures, and monitoring safeguards ensures consistency. (ii) As indicated by the exclusion of Child-Pugh C cirrhosis or severe cardiopulmonary disease, the initial safety assessment is focused on typical ambulatory candidates. Nevertheless, further investigation into higher-risk cohorts is considered essential. (iii) The psychoactive effects of esketamine, despite being mitigated by the administration of a standard dose of midazolam before the commencement of the trial and by assessors who were unaware of the treatment allocation, may compromise the integrity of the blinding process.

### 5.3 Future and direction

It is recommended that future research explore the impact of esketamine on long-term recovery metrics, cost-effectiveness, and its utility in comorbid populations (eg. advanced liver disease or severe sleep apnea). Beyond its well-established analgesic properties, esketamine has demonstrated significant efficacy in the treatment of depressive disorders (Martinotti et al., 2022; Di Vincenzo et al., 2024; Rosso et al., 2025). The esketamine-based analgesic regimen examined in this study has the potential to offer dual therapeutic benefits, effectively addressing perioperative analgesic requirements while potentially ameliorating peri-procedural emotional distress. Further investigation into the psychotropic benefits of perioperative esketamine in oncology patients is a promising avenue for future research.

We should also explore combining esketamine with targeted nerve blocks to achieve truly opioid-free ablation while testing this approach in similar outpatient procedures like kidney tumor (Dubut et al., 2016), lung tumor (Wang et al., 2025), or uterine fibroids (Yan et al., 2024) treatments. These concrete steps will determine if esketamine can become a safe, scalable solution for reducing/avoiding opioid reliance during minimally invasive tumor therapies.

Although preclinical evidence indicates that μ-opioid receptor (MOR) activation might promote tumor progression in hepatocellular carcinoma (Li et al., 2019), the potential survival benefits of an opioid-free anesthetic regime in this context remain speculative and require prospective clinical validation.

Additionally, artificial intelligence and machine learning tools could enable precise predictions of analgesic requirements during anesthesia for these outpatient procedures (Salama et al., 2024), optimizing esketamine dosing through real-time pain classification and risk stratification.

## 6 Conclusion

The objective of this trial is to provide Level I evidence to inform the selection of analgesics for ambulatory percutaneous liver ablation procedures. Should esketamine prove to be a more efficacious agent in terms of respiratory safety, it has the potential to establish HHNB-esketamine as a new standard for same-day discharge pathways in ASCs, thereby aligning analgesic efficacy with enhanced recovery after surgery (ERAS).
